# Natural killer cells: the immune frontline against circulating tumor cells

**DOI:** 10.1186/s13046-025-03375-x

**Published:** 2025-04-10

**Authors:** Doryan Masmoudi, Martin Villalba, Catherine Alix-Panabières

**Affiliations:** 1 Laboratory of Rare Circulating Human Cells, University Medical Center of Montpellier, Montpellier, France; 2 IRMB, Univ Montpellier, INSERM, CHU Montpellier, CNRS, Montpellier, France; 3 CREEC/CANECEV, MIVEGEC (CREES), University of Montpellier, CNRS, Montpellier, IRD France; 4 European Liquid Biopsy Society (ELBS), Hamburg, Germany; 5 LCCRH, Site Unique de Biologie (SUB), 641, Avenue du Doyen Gaston Giraud, Montpellier, 34093 France

**Keywords:** Cancer, Liquid Biopsy, Circulating tumor cells, Natural killer cells, Biomarkers

## Abstract

Natural killer (NK) play a key role in controlling tumor dissemination by mediating cytotoxicity towards cancer cells without the need of education. These cells are pivotal in eliminating circulating tumor cells (CTCs) from the bloodstream, thus limiting cancer spread and metastasis. However, aggressive CTCs can evade NK cell surveillance, facilitating tumor growth at distant sites. In this review, we first discuss the biology of NK cells, focusing on their functions within the tumor microenvironment (TME), the lymphatic system, and circulation. We then examine the immune evasion mechanisms employed by cancer cells to inhibit NK cell activity, including the upregulation of inhibitory receptors. Finally, we explore the clinical implications of monitoring circulating biomarkers, such as NK cells and CTCs, for therapeutic decision-making and emphasize the need to enhance NK cell-based therapies by overcoming immune escape mechanisms.

## Introduction

Cancer metastasis formation can be described as a series of steps in which tumor cells detach from the primary tumor, enter the bloodstream (i.e., circulating tumor cells, CTCs) and colonize distant sites [1]. Metastatic disease is the most frequent cause of patient death [2]. Moreover, most patients with metastatic cancer respond only transiently or not at all to conventional treatments. A more detailed elucidation of the immune evasion mechanisms of CTCs, which are at the core of the metastatic cascade, will contribute to the development of new therapies. It has been known for several decades that natural killer (NK) cells play a prominent role in controlling metastasis formation and eliminating CTCs [3]. These immune cells mediate cytotoxicity of stressed or malignant cells, independently of major histocompatibility complex (MHC) component expression [4]. Although NK cells have a low capacity to infiltrate solid tumors, their presence is often associated with better prognosis [5]. Their low infiltration in the tumor microenvironment (TME) and their compromised action in the lymphatic system are the results of a complex immunosuppressive communication network that involves other immune cell types, cancer cells and the physical–chemical characteristics of the solid tumor. However, little is known about the bloodstream environment and the conditions underlying NK cell immunosurveillance evasion by some tumor cells. At the dawn of the development of NK cell-based therapies and the advent of liquid biopsy as a diagnostic tool, the aim of this review is to promote studies on how CTCs evade NK cell surveillance. After an overview of NK cell biology, we describe the immune evasion mechanisms in the TME. We then summarize the information obtained by the analysis of circulating NK cells, as a liquid biopsy analyte. We also discuss some mechanisms of CTC evasion and lastly, we present some clinical aspects of the combined analysis of CTCs and NK cells.

## NK cell biology

NK cells are lymphocytes belonging to the innate immune system. Unlike B and T lymphocytes, in NK cells receptor-encoding genes do not undergo somatic recombination. NK cells were first identified in 1975 when Herberman et al*.* observed that some murine lymphocytes displayed MHC-I-independent cytotoxicity against allogeneic tumors, differently from cytotoxic T lymphocytes [6]. Later, NK cells were recognized as part of another lymphoid lineage and as cells that mediate cytotoxicity through granule and cytokine secretion and are regulated by complex cellular interactions [7]. This evolutionarily-acquired cytotoxicity is the result of a long process that is finely regulated in order to distinguish between target cells and “self” cells. This was established by Ljunggren, Hans-Gustaf, and Klas Kärre's “missing self” theory in 1990, explaining that NK cells are able to distinguish abnormal cells, such as tumor or infected cells, by the absence of certain molecules, mainly class I MHC molecules, on their surface [8]. NK cells do not specifically recognize tumor cells, but they do detect the absence of MHC-I molecules. This dynamic equilibrium implies a balance between a variety of activating and inhibiting receptors. Inhibitory receptors on NK cells, such as some KIR (Killer Immunoglobulin-like Receptors) and LIR (Leukocyte Immunoglobulin-like Receptors), mainly recognize MHC-I molecules present on the surface of normal cells [9, 10]. The KIRs' main ligands are Human Leukocyte Antigen class I molecules (HLA-A, HLA-B, HLA-C, HLA-G). Still based on the “missing self” principle, healthy cells express MHC-I molecules, which interact with inhibitory KIRs (KIR2DL1, KIR2DL2, KIR2DL3, KIR3DL1, KIR3DL2) inhibiting NK cells and preventing their cytotoxicity. Activating KIRs (KIR2DS1, KIR2DS2, KIR3DS1, KIR2DS4) bind to specific ligands (modified MHC-I, stress proteins, etc.), triggering a cascade of signals via adaptor proteins such as DAP12, which carries ITAM motifs. NK cell activation leads to cytokine secretion (IFN-γ, TNF-α) and cytotoxicity via granzyme and perforin release, leading to apoptosis of target cells [11]. Unlike MHC genes, KIRs are organized variably among individuals, creating major functional diversity [12]. This polymorphism plays a key role in the immunosurveillance efficiency of NK cells, influencing their ability to fight cancer [13]. Some NK cell subtypes express LIRs, such as LILRB1 [14, 15]. These receptors belong to the immunoglobulin superfamily and play a crucial role in modulating innate and adaptive immune responses [16]. In particular, they play a key role in inhibiting NK responses by interacting with HLA-G and HLA-I [15]. In addition, NKs express NKG2A, an inhibitory receptor belonging to the C-lectin receptor family [17, 18]. NKG2A and its CD94 heterodimer (NKG2A/CD94) interact with HLA-E on the surface of target cells, inducing an inhibitory signal in the NK cell via its ITIM (Immunoreceptor Tyrosine-based Inhibitory Motif) domain [19].

The TIGIT receptor (T-cell immunoreceptor with Ig and ITIM domains) is primarily recognized for its inhibitory functions on NK cells, and when engaged by its ligand CD155 (also known as poliovirus receptor, PVR), TIGIT exerts an inhibitory effect via its ITIM domains [20, 21]. Its expression on NKs, although activated, induces a decrease in their activity. In particular, Hasan et al. have shown that TIGIT expression on activated NK cells is associated with better initial antitumor activity, leading nevertheless to functional decline [22]. TIGIT expression on NK cells also influences the dendritic cell environment [23]. When they interact with TIGIT, dendritic cells acquire a mature, immunoregulatory profile. These regulatory dendritic cells (DCregs) are particularly effective at inducing immune tolerance, thereby suppressing T cell activation.

NK-activating receptors, such as NKG2D, NKp30, NKp44, NKp46, 2B4 and DNAM-1 detect molecules mainly expressed by stressed, tumor or infected cells. Activation of these activating receptors stimulates NK cytotoxic activity trough secretion of perforin and granzymes and the production of pro-inflammatory cytokines, such as IFN-γ [9, 10]. For example, the NKG2D receptor interacts with the ligands MICA/B and ULBP, which are often overexpressed on tumor cells in response to stress [24, 25]. DNAM-1 binds two main ligands also expressed on the surface of tumor cells or virus-infected cells, CD155 (poliovirus receptor, PVR) and CD112 (nectin-2) [26, 27]. The 2B4 receptor (also known as CD244) mainly recognizes a ligand called CD48 [28]. Moreover, the interaction between 2B4 and CD48 plays a central role in the regulation of immune responses by stimulating the proliferation of NK and T cells [29]. The balance between activating and inhibiting receptors on NK cells determines their ability to respond appropriately to infected or tumor cells, by regulating their activation or inhibition according to the signals received.

NK cells are found in all lymphoid and non-lymphoid tissues where thy carry out their sentinel function. Their role and cytotoxic activity are intimately linked to their level of differentiation and maturity. In humans, NK cells are classified in two main subclasses: CD56^dim^ and CD56 ^bright^. The majority of NK cells (~ 90%) in blood have low CD56 expression (CD56^dim^) but high Fcγ receptor III (CD16^bright^) expression. The other (~ 10%) NK cells strongly express CD56 (CD56^bright^) and weakly CD16 (CD16^dim^) [30]. Conversely, CD56^bright^ NK cells accumulate in the lymph node microenvironment where they contribute to immunoregulation [31]. Very early on, CD56^dim^ CD16^bright^ NK cells were recognized as the most cytotoxic NK cell subtype [32]. In 2024, Rebuffet et al*.* carried out a single-cell transcriptomic analysis of NK cells from 718 healthy donors to identify different NK cell subgroups. This analysis allowed classifying NK cells in three groups (NK1,NK2,NK3) that were further differentiated into six subgroups, according to their transcriptomic profile [33]. Moreover, NK subtype distribution varies in function of the tumor type. Similarly, most CD56^dim^ CD16^bright^ NK cells are found in peripheral blood and spleen, whereas CD56^bright^ CD16^dim^ NK cells are mostly observed in lymph nodes and tonsils and lacks perforin [34]. Although CD56^bright^ NK cells are less cytotoxic, they secrete a significant number of proinflammatory cytokines, such as interferon (IFN)-γ and tumor necrosis factor (TNF)-α [35]. The relationship between these NK cell phenotypes is still controversial, but data on telomere length suggest that CD56^dim^ cells represent a more mature phenotype [36]. Thus, the NK cells present in secondary lymphoid organs (e.g. lymph nodes, spleen, tonsils, appendix) would represent the final maturation and self-tolerance stages.

NK cell cytotoxicity is mediated through the expression of specific receptors, but is also regulated by the presence and concentration in the microenvironment of cytokines and some immune cell types (e.g. T cells, macrophages and dendritic cells). Since 1999, it is known that dendritic cells can promote NK cell cytotoxicity and IFN-γ production via cell-to-cell contacts [37]. NK cells produce IFN-γ through paracrine and autocrine mechanisms. IFN-γ specifically increases MHC-I expression on target cells, allowing CD8^+^ and CD4^+^ T cells to recognize them more effectively [38]. IFN-γ also enhances dendritic cell maturation and their ability to deliver antigens to T cells [39] and improves NK cell cytotoxic activity by stimulating the release of perforin and granzymes. Hence, IFN-γ is a crucial mediator of NK cell immune activity directly by affecting target cells and indirectly by coordinating the function of other immune cell types [40]. On the other hand, NK cell functions are negatively regulated by some cytokines, such as transforming growth factor (TGF)-β [41].

### NK cell role in cancer immunosurveillance

#### NK cells in the tumor microenvironment

During tumor development, cancer cells can escape NK cell-mediated lysis through suppression of NK cell function or by harboring a low immunogenicity phenotype.

**Fig. 1:**
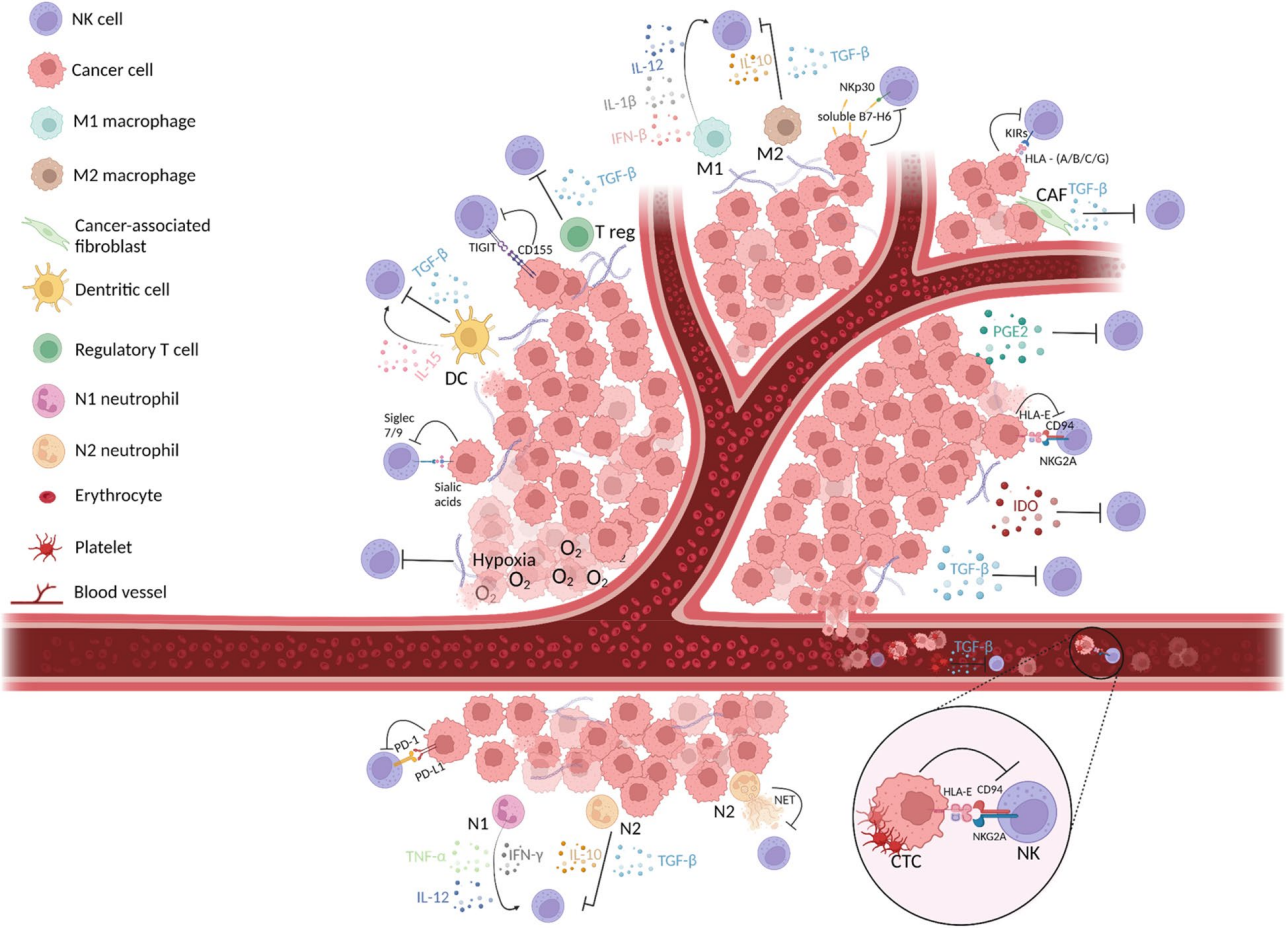
Cancer cell immune escape from NK cells during metastasis formation. Schematic representation of the interactions between the TME and NK cells and of some immune evasion mechanisms. The cytotoxic capacity of NK cells can be inhibited by cytokines, such as TGF-β and IL-10, enzymes, such as IDO, or molecules, such as prostaglandin E2, produced by TME cells. Conversely, NK cells activity is promoted by other cytokines present in the TME and secreted by immune cells, such as IL-15, IL-1β, IL-12 and IFN-β. Tumor cell escape is also mediated by direct interactions with NK inhibitory receptors such as NKG2A, PD1, TIGIT, Siglec 7 and 9, and certain KIRS (KIR2DL1, KIR2DL2, KIR2DL3, KIR3DL1, KIR3DL2). In addition, certain metalloproteases release soluble ligands, such as B7-H6, which bind to the NKp30 activator receptor, thus contributing to NK exhaustion. During tumor progression, some cells detach from the tumor mass and enter the bloodstream. Some CTCs escape NK cell immunosurveillance, particularly by interacting with platelets. For instance, the presence of the platelet-derived *RGS18* mRNA on CTCs promote the overexpression of the HLA-E complex. Binding of this complex to the NKG2A/CD94 receptor induces phosphorylation of the tyrosine residues of its ITIM domains that then recruit phosphatases to block NK cell cytotoxic activity. TGF-β secretion by platelets also allows CTCs to escape lysis. *Created with BioRender.com. Abbreviations*: CTCs, Circulating Tumor Cells; HLA, human leukocyte antigen; IDO, indoleamine 2,3-dioxygenase; IL, interleukin; IFN-β, interferon-β; NET, neutrophil extracellular trap; NKs, natural killers; TGF-β, transforming growth factor β; TME, tumor microenvironnement; IFN-β, interferon-β

The inflammatory TME is characterized by a complex network of interactions between tumor cells, tumor stem cells, immune cells, extracellular matrix, blood vessels, fibroblasts and some signaling molecules that influence cancer progression (Fig. 1). Macrophages are the most prominent immune cell type in the TME. Depending on the various signals present in the TME (cytokines, growth factors, cell interactions), macrophages can polarize into two types (M1 or M2). M1 and M2 macrophages display different functional phenotypes, resulting in significant differences in marker expression, biological functions and secretion of pro- or anti-inflammatory mediators. In the TME, macrophage polarization balance tends towards the M2 anti-inflammatory phenotype (i.e. tumor-associated macrophages) [42]. M2 macrophages promote cancer progression, notably by suppressing the immune response. Moreover, they secrete cytokines, such as TGF-β, that induce a reduction of the expression of NKp30 and NKG2D, two activating NK cell receptors fundamental for cancer cell clearance [43]. Many studies highlighted the role of M2 macrophages in TGF-β secretion [44–47]. M2 macrophages also secrete interleukin (IL)−10 that at high doses, affects the ability of NK cells to respond to activating cytokines, such as IL-15 and IL-18 [48, 49]. IL-10 inhibitory activity towards NK cells has been reported in patients with gastric cancer [50]. To escape lysis, M2 macrophages can express HLA-E that negatively regulates NK cells and induces the secretion of immunosuppressive cytokines by binding to the inhibitory receptor NKG2A/CD94 on NK cells [51, 52].

On the other hand, M1 polarization represents a pro-inflammatory state induced by signals such as IFN-γ or TNF-α, and characterized by high production of pro-inflammatory cytokines. M1 macrophages can activate NK cell cytotoxicity through a complex network of soluble mediators, such as IL-23, IFN-β and IL-1β [53]. Interestingly, NK cells activate macrophages via secretion of IFN-γ, TNF-α and granulocyte–macrophage colony-stimulating factor, thus creating a kind of feedback loop [54, 55]. The pro-inflammatory cytokines secreted by M1 macrophages (e.g. IL-12 and IL-15) [56, 57] boost the anti-tumor immune response by promoting IFN-γ secretion, cytotoxic activity and NK cell maturation and proliferation [58, 59]. Moreover, macrophages can express ligands of the activating receptor NKG2D to create a positive feedback loop that promotes their polarization into the M1 type and increases NK cell cytotoxicity [60]. In conclusion, the relationship between NK cells and macrophages in the TME is dynamic and complex. This relationship can have an immunosuppressive and pro-tumor effect or an anti-tumor effect depending on the macrophage polarization (M1 or M2) and also the tumor stage and location [61].

Macrophages are not the only immune cell type that can inhibit NK cell cytotoxicity against cancer cells in the TME. T regulatory cells (Tregs) play a central role in immunosuppression within the tumor microenvironment (TME), limiting the efficacy of NK cells in their anti-tumor response [62]. T reg cells are a T-cell subpopulation (FOXP3^+^CD25^+^CD4^+^) involved in immunomodulation and self-tolerance. T reg cells compete with NK cells for the binding to IL-2, involved in NK cell activation and proliferation [63]. In patients with gastrointestinal stromal tumor, T reg cells affect NK cell activity, and thus tumor expansion, by reducing NKG2D expression [64]. T reg cells can also suppress antibody-dependent cellular cytotoxicity, one of the mechanisms of action of NK cells, in patients treated with cetuximab [65]. It is well known that Treg can secrete immunosuppressive cytokines, notably TGF-β and IL-10 [66–69]. These cytokines hinder dendritic cells (DCs) maturation, reducing their ability to effectively activate NKs [62]. In addition, Tregs may interact directly with NKs via contact molecules such as CTLA-4 or PD-1 [62, 69–71]. It even appears that a subpopulation of LAP^+^ Foxp3^−^ T cells may exert even greater immunosuppressive activity in the context of colorectal cancer [69]. Lainé et al*.* also showed in breast cancer models that T reg cells activate TGF-β that contributes to decrease NK cell activity [72].

DCs and NK cells play a crucial role in immune surveillance and anti-tumor response [73]. Their bidirectional cross-talk influences both innate and adaptive immune responses. However, in the TME, this relationship is often altered, favoring immune evasion of cancer cells. Mature DCs secrete IL-12, IL-15 and IL-18, which stimulate NK proliferation and cytotoxicity, leading to increased expression of their activator receptors (NKG2D, NKp30, NKp46) and IFN-γ production [37, 74]. DCs trans-present IL-15 via IL-15Rα, promoting NK survival and expansion [74, 75]. On the other hand, NKs eliminate immature DCs (via NKp30) and promote DC maturation via IFN-γ and TNF-α secretion [76]. Nevertheless, tumor cells and immunosuppressive cells (Treg and M2 macrophages) induce an immunosuppressive phenotype in DCs. Firstly, DCs remain immature under the influence of TGF-β and IL-10. They weakly express co-stimulatory molecules (CD80, CD86, CD40), reducing their capacity to activate NKs [77]. Capture of tumor antigens is often inefficient or misdirected, preventing their optimal presentation via MHC-I and MHC-II [77]. In addition, increased PD-1 expression is found in certain DC populations, reducing NK cell activation [78]. DCs in the TME will also inhibit NK activity through the secretion of cytokines and immunosuppressive enzymes (TGF-β, IL-10, indoleamine 2,3-dioxygenase) that inhibit NK proliferation and cytotoxicity [79–81]. TGF-β secretion also leads to the differentiation and expansion of Tregs [79]. Ahluwalia et al*.* have notably shown that DCs in NSCLC TME show signs of dysfunction and have a reduced capacity to produce NK-stimulating cytokines [82]. In the context of this NK-DC interaction, NKs also play an important role in modulating the immune environment. Specifically, NKs stimulate the recruitment of classical type 1 dendritic cells (cDC1) into the TME via the secretion of IL12 [83]. Once recruited to the TME, cDC1s are able to capture tumor antigens and present them to T lymphocytes. The NK-DC axis is crucial to the anti-tumor immune response via tumor antigen presentation to T cells [84]. The relationship between NK cells and DCs in TME is complex and plays a crucial role in regulating the antitumor immune response. This interaction is often impaired in TME, where tumor cells and immunosuppressive signals can reduce the efficacy of both NK and DC.

PMNs (polymorphonuclear neutrophils), or neutrophils, play an ambivalent role in the tumor microenvironment (TME), and their interaction with NKs can have complex effects on the antitumor immune response [85]. Normally associated with acute inflammatory responses, they show significant plasticity in the TME and can adopt both pro- and anti-tumoral roles, depending on the signals they receive in the microenvironment [86]. Their plasticity and ability to modulate the immune environment make them key players in tumor dynamics. Neutrophils in TME can be polarized into two distinct subpopulations, called N1 and N2, notably under the influence of the cytokine TGF-β [87, 88]. N1 neutrophils are key players in anti-tumor immunity, stimulating immune responses through the secretion of TNF-α, IL-12 and IFN-γ, exerting direct cytotoxicity via protease release, and limiting tumor progression by inhibiting angiogenesis [89–92]. However, under the influence of the tumor microenvironment, these neutrophils can be converted into a pro-tumor phenotype [89, 93]. Polarization towards an N2 (immunosuppressive) type increases the production of immunosuppressive cytokines, such as IL-10 and TGF-β, thus affecting NK activity [87]. Additionally, TME neutrophils strongly express PD-L1, which interacts with PD-1 expressed by NK cells, leading to a reduction in their cytotoxicity and IFN-γ production [94]. This interaction inhibits activation of the NKp46 and NKG2D receptors, limiting their ability to eliminate tumor cells [94]. We also know that neutrophils, via their extracellular vesicles, can negatively influence NK activity, contributing to immunosuppression and tumor progression [95]. Furthermore, it appears that neutrophils mobilized by primary tumors have the capacity to prevent clearance of tumor cells by NKs in the initial sites of dissemination, while facilitating tumor cell extravasation by increasing endothelial permeability [96]. On the other hand, NKs play a crucial role in controlling neutrophil tumor activity. Their absence in murine models promotes the conversion of neutrophils into a pro-tumor population [97]. Teijeira et al. have demonstrated the production of neutrophil extracellular traps (NETs, complex structures made up of nuclear DNA and cytoplasmic proteins) by neutrophils present in TME [98]. They form a physical barrier that prevents direct access of NK cells to tumor cells [98].

Cancer-associated fibroblasts are another prominent cell type in the TME. This highly heterogeneous cell population has roles in angiogenesis, extracellular matrix remodeling, cancer cell survival and immune response modulation [99]. In co-cultures of NK cells with melanoma-derived fibroblasts, prostaglandin E2 secretion by fibroblasts modulate the expression of the NKp44, NKp30 and DNAM-1 receptors [100], impairing NK cell ability to kill cancer cells. Moreover, the secretion of active matrix metalloproteinases by cancer-associated fibroblasts decrease the expression of MIC-A and MIC-B (NKG2D ligands) on the melanoma cell surface, leading to impaired NK cell activity [101]. Similarly, hepatocellular carcinoma-derived fibroblasts negatively modulate NK cell activity through secretion of prostaglandin E2 and indoleamine 2,3-dioxygenase (IDO) [102].

Cancer cells also can reduce NK cell activity. For example, expression of NKp30, NKp44, and NKG2D is decreased when NK cells are co-cultured with melanoma cells that secrete enzymes, cytokines and growth factors [103]. Moreover, during tumor development, glycosylation of cancer cell surface proteins is altered. In many cancers, this leads to hypersialylation and to aberrant presence of the sialic acid N-acetylneuraminic acid on cell surface glycans [104]. This promotes cancer cell binding to the NK cell inhibitory receptors Siglec 7 and 9, resulting in NK cell inactivation [105, 106]. Cancer cells also secrete TGF-β in an inactive form. Schlecker, Eva et al*.* also demonstrated that tumor cells release a soluble form of B7-H6 through the action of metalloproteinases. The interaction between soluble B7-H6 and NKp30 on NK cells could prevent optimal activation of the latter, diminishing their capacity to destroy tumor cells [107]. They also highlight the fact that B7-H6 is frequently detected in the serum of patients with malignant melanoma [107].

TME homeostasis disruption leads to a hypoxic environment, and consequently, the expression of NK cell-activating receptors, such as NKp46, NKp30, NKp44, and NKG2D, is decreased [108]. Besides this direct effect on NK cell activity, hypoxia causes the downregulation of some ligands of the NKG2D receptor (e.g. MIC-A) on the surface of osteosarcoma cells, potentially reducing NK cell cytotoxic activity [109]. Baginska et al. demonstrated that in vitro, in conditions of hypoxia, breast cancer cells escape lysis by NK cells by activating autophagy to degrade NK cell-derived granzyme B [110]. These findings show that hypoxia-induced stress negatively regulates NK cell anti-cancer immunosurveillance, thus promoting cancer development.

Metabolic competition for glucose also is a limiting factor for NK cell anti-cancer immunosurveillance. Upon lymphocyte activation, glucose consumption is increased to meet their strong energetic and biosynthetic demands [111]. Cong et al*.* demonstrated that during lung cancer development/progression, NK cells gradually become dysfunctional and lose their anti-cancer activity due to aberrant expression of the enzyme fructose-1,6-bisphosphatase, which prevents glycolysis. In agreement, NK cell activity can be restored by blocking this enzyme [112]. NK cells that cannot sustain high glycolysis have reduced activity [113]. In activated NK cells, the transcription factor SREBP is required for the elevated glycolysis and oxidative phosphorylation and its inhibition hinders IFN-γ production and NK cell cytotoxic activity [114]. Interestingly, in some cancer types (*e.g.* colorectal, gastric cancer), SREBP inhibitors, such as 27-hydroxycholesterol, are strongly expressed in the TME [115–117].

NK cell anti-tumor immunosurveillance is also regulated by the expression of chemokines and their receptors [118]. For instance, in melanoma, the chemokine CXCL10 increases NK cell infiltration in the TME and reduces tumor growth in vitro [119]. However, in melanoma cells that strongly express inducible nitric oxide synthase, CXCL10 expression is reduced [120]. Moreover, in a study on 182 patients with pancreatic adenocarcinoma, CXCL10 expression correlated with better prognosis [121]. Conversely, CXCL10 overexpression in hepatocellular carcinoma has been correlated with poorer overall survival [122]. Therefore, CXCL10 plays a dual role in the TME by promoting either the antitumor immunosurveillance or immunosuppression and tumor progression, in function of the tumor type and specific TME characteristics.

The chemokine CXCL9 attracts cells that express the CXCR3 receptor, such as NK cells, to the tumor site [123]. Its overexpression in the TME is a good prognostic factor in intrahepatic cholangiocarcinoma [124], early-stage lung adenocarcinoma [125], estrogen receptor-negative breast cancer [126] and endometrial cancer [127]. However, data on CXCL9 role in tumor progression are contradictory [123]. Nevertheless, CXCL9 is implicated in NK cell recruitment and immunosurveillance in the TME.

The classification of NKs into two main subpopulations based on their level of CD56 marker expression is much less marked and more altered in the tumor microenvironment due to tumor signals and immunosuppression (hypoxia, immunosuppressive cytokines and immunomodulatory cells). NK cells infiltrating liver tumors often have a high expression of NKG2A/CD94 and this is associated with a poor prognosis [128]. In human cervical carcinoma, we also find upregulation of CD94/NKG2A inhibitory receptors in NK cells [129]. These tumor-infiltrating NK cells (TINKs) have an altered phenotype with reduced activation markers and often express more inhibitory receptors [130]. These TINKs have an altered phenotype with reduced activation markers and often express more inhibitory receptors. This is emphasized by Bruno et al*.* who explored the transcriptional profile of TINK cells in various types of cancer [131]. RNA sequencing data from human tumors identified distinct NK subpopulations illustrating a large proportion of TINKs display an inhibited phenotype, with overexpression of inhibitory receptors (e.g. NKG2A, TIGIT, KIRs). In particular, they found an enrichment of dysfunctional and stressed CD56^bright^ TINKs, as well as cytotoxic effector CD56^dim^. They point out that these TINKS have a clear signature of tissue residence, but still share the dominant classification of CD56^bright^ and CD56^dim^ NK cells found in blood [131].

Given the critical role of NK cells in cancer cell recognition and elimination, their infiltration into the TME is frequently associated with a favorable prognosis. NK cells contribute to enhance the anti-tumor immunity by directly lysing cancer cells and secreting immunostimulatory cytokines, such as IFN-γ. They also play a crucial role in limiting metastatic spread. In a meta-analysis of 53 studies on NK cell infiltration in solid tumors and its correlation with patient survival outcomes, Nersesian et al*.* concluded that higher levels of NK cell infiltration in the tumor are associated with reduced risk of mortality [132]. However, their activity is significantly hampered by the TME through immunosuppressive signals and factors, such as TGF-β, pro-inflammatory cytokines and regulatory immune cells. Together, these elements alter NK cell function, reducing their ability to recognize and eliminate cancer cells [130, 133, 134]. In addition to soluble immunosuppressive factors, tumor cells can directly impair NK cell function by exploiting inhibitory immune checkpoints. Among these, NKG2A and PD-1 have attracted increasing interest due to their role in impairing NK activity in the tumor microenvironment. HLA-E, often overexpressed on cancer cells, binds to the inhibitory receptor NKG2A, leading to functional depletion and reduced cytotoxicity of NKs. Similarly, PD-1 expression on NK cells, observed in several types of cancer, can be induced by prolonged exposure to the tumor microenvironment and further repress their antitumor response by interacting with PD-L1-expressing tumor cells. The clinical importance of these pathways is highlighted by emerging therapeutic strategies aimed at blocking NKG2A (*e.g.* monalizumab) or the PD-1/PD-L1 axis, which have shown promising results in restoring NK and T cell tumor surveillance [135–137]. Studying the expression profiles and functional consequences of these checkpoints on tumor-infiltrating NK cells could provide valuable insights for the development of new immunotherapeutic approaches.

#### NK cells in the lymphatic system

The lymphatic system is a vascular network parallel to the blood system, essential for maintaining body homeostasis. It is made up of lymphatic vessels, lymph nodes and lymphoid organs such as the spleen and thymus. Its main role is to ensure the drainage of interstitial fluids, facilitating the return of liquids to the bloodstream. It also plays a key role in immunity, transporting immune cells and enabling pathogen recognition within the lymph nodes. In addition to these immune and circulatory functions, it is involved in the absorption of dietary lipids via the intestinal chyliferous tract [138]. The lymphatic system plays a crucial role in immunity, monitoring, transporting and activating immune cells. It filters antigens through the lymph nodes, where dendritic cells activate B and T lymphocytes. These cells then patrol the body to detect and respond to threats. In addition to coordinating the innate and adaptive immune response, it participates in immune memory and fluid drainage, regulating inflammation and optimizing the body's defenses [139]. It also plays a role in peripheral immune tolerance, preventing lymphocyte reactivity towards the body's own cells.

Lymphatic NK cells are a distinct subpopulation of circulating NKs, and play a crucial role in immunosurveillance and the orchestration of immune responses. Present in secondary lymphoid organs such as lymph nodes, spleen and Peyer's patches, they differ from blood NKs in their phenotype and functionality. Unlike circulating NK cells in peripheral blood, lymphatic NKs express fewer activating and inhibitory receptors, notably KIRs, suggesting that NKs in secondary lymphoid organs play an immunomodulatory rather than cytotoxic role under basal conditions [140]. The existence of functional heterogeneity of NK cells according to their location is further illustrated by the existence of a CD69^+^CXCR6^+^ subpopulation that is not found in the bloodstream, supporting the hypothesis that this NK cell population is adapted to the lymphoid microenvironment and is therefore resident [141].

During the immune response, NK cells are actively recruited to the lymph nodes via the expression of chemokines such as CCL5 and CXCL10 [142]. Once in this environment, they produce IFN-γ, a key cytokine for dendritic cell activation and the polarization of naive CD4 + T cells towards a Th1 profile [142]. They thus interact closely with dendritic cells and T lymphocytes, influencing the activation and polarization of adaptive responses. Real-time imaging reveals that NKs migrate rapidly through lymphoid structures and preferentially target certain areas, such as the T-zone of lymph nodes, where they can modulate the immune response [143]. Bajénoff et al*.* have also demonstrated that NK cells in lymph nodes are not simply circulating killer cells, but play a crucial role as immune mediators [143]. When an inflammatory stimulus is detected (pathogenic or tumoral), NKs establish prolonged contacts with mature dendritic cells (mDCs), which provide activating signals via IL-12 and IL-18. This activation induces high IFN-γ production and promotes differentiation of naive CD4^+^ T cells [143]. Under basal conditions, NK cells of the lymphatic system play an essential immunomodulatory rather than strictly cytotoxic role. Mainly of the CD56^bright^ phenotype, they actively migrate into lymph nodes, interact with dendritic cells and contribute to the orchestration of the immune response via the production of cytokines, notably IFN-γ. They thus participate in the polarization of Th1 responses and the activation of antigen-presenting cells, reinforcing immune surveillance without exerting an immediate killing function.

During carcinogenesis, tumor cells can secrete lymphangiogenic factors such as VEGF-C and VEGF-D, promoting tumor lymphangiogenesis [144–146]. As a result, tumor cells are able to penetrate the lymphatic system, dilate collecting lymphatic vessels and disseminate to distant lymph nodes and organs [147]. Tumor cells follow much the same pattern of colonization and dissemination in the lymphatic system as they do in the bloodstream. Cancer cells circulate in the lymph and then reach the sentinel lymph nodes. The most aggressive resist mechanical and immune constraints by aggregating, modulating the microenvironment and expressing immune checkpoints [148, 149]. Once in the lymph nodes, tumor cells can establish metastatic niches by interacting with the lymph node's immune and stromal microenvironment, reprogramming local immune cells (M2 macrophages, regulatory T cells) and modulating the dendritic cell response to prevent effective activation of the immune system [150–152]. Metastatic cancer cells can then enter the bloodstream (secondary hematogenesis) via the post-capillary vessels of the lymph nodes, migrate to other organs and exploit lymphatic drainage to reach other lymph nodes and amplify their dissemination [153].

For the past ten years or so, we have known that the lymphatic system plays a role in tumor development through immunomodulatory mechanisms. Consequently, the phenotypic heterogeneity of NKs present in the lymphatic system, with a generally less cytotoxic and more immunomodulatory phenotype, may play a more controversial role than in the bloodstream. Since 1986, we have known that NK cell activity in lymph nodes, particularly cervical lymph nodes, is significantly reduced in the presence of metastasis [154]. We report the presence of CD16⁺NKG2A^high^ NKs in lymph nodes draining breast cancer [155]. High NKG2A expression could be exploited by HLA-E-expressing cancer cells, enabling them to evade NK immune surveillance [155]. Moreover, Tregs recruited and educated by the tumor infiltrate draining lymph nodes and suppress NK cell activity, reducing their ability to eliminate disseminated tumor cells through TGF-β and IL-10 secretion and enhanced PD-L1 expression [156]. Reticker-Flynn et al*.* shed light on the fact that tumor cells in lymph nodes induce immune tolerance that specifically inhibits NK cell activity through upregulation of MHC-I and PD-L1, thus facilitating tumor survival and the spread of distant metastases [157].

However, it has been shown that a greater presence of NKs in the largest lymph nodes is associated with a better prognosis in patients with stage II colon cancer [158].

We observe that in the lymphatic system, cancer cells can escape the surveillance of NKs by modifying the lymphatic microenvironment to create an immunosuppressive environment. In addition, the presence of Tregs in lymph nodes and PD-1 expression by lymphatic endothelial cells may also inhibit the cytotoxic action of NKs and promote metastasis [159, 160]. However, there is a lack of studies on immunomodulatory NK populations and their role in tumor dissemination.

#### NK cells in the bloodstream

Solid tumors release cancer cells into the bloodstream. The most aggressive CTCs that survive the mechanical stress and immune system attacks may form secondary tumor at new sites. In this context, NK cells contribute to tumor growth control by infiltrating the TME and by lysing CTCs to limit their dissemination [161–163]. Therefore, liquid biopsy, a tool for detecting and monitoring cancer progress, is relevant for the analysis of circulating NK cells [164].

In 1993, a study showed for the first time that the mean lytic activity of NK cells isolated from the blood of 24 patient with prostate cancer and co-cultured with commercial cancer cell lines decreased with the tumor stage [165]. The authors suggested the potential utility of assessing circulating NK cells as a tool for determining cancer stage. Since then, NK cell phenotypic enumeration and characterization have provided a wealth of information on tumor progression and patient prognosis, highlighting their fundamental role in controlling tumor dissemination.

Particularly, decreased expression of NKp30, NKp46, NKG2D, and DNAM-1 has been associated with gastric cancer progression [166]. NKp46 expression on the surface of CD56^dim^ CD16^bright^ NK cells predicts better survival in patients with non-small cell lung cancer (NSCLC) [167]. In addition, NK cell function (degranulation, IFN-γ production and NKG2D expression) is progressively altered during pancreatic cancer development. In a prospective analysis, this impairment was correlated with cancer recurrence and mortality [168]. In a retrospective study of patients with metastatic prostate cancer, the presence of specific markers, such as NKp30 and NKp46, on NK cell surface was correlated with improved overall survival [169]. These findings suggest that NK cell presence and the expression of some activation receptors positively influence the patient prognosis and tumor progression control. Interestingly, some studies indicate that high NK cell count in gastric cancer is associated with poor patient survival [170]. Expression of inhibitory receptors, such as NKG2A/CD94, in liver cancer also is a predictive factor of poor prognosis [128]. Thus, the phenotype of circulating NK cells appears to be a prognostic factor. In patients with colorectal cancer, co-culture experiments showed that degranulation and IFN-γ production activity are reduced in both tumor-associated NK cells and peripheral NK cells [171]. This altered function has been observed also in circulating NK cells from patients with advanced breast cancer. As a result, the proportion of immature and non-cytotoxic NK cell subpopulations is increased [172]. This NK cell phenotype plasticity is strongly linked to the TME. Campos-Mora et al*.* found that some circulating NK cells can incorporate solid tumor markers via trogocytosis. Nevertheless, the authors suggested that the number of NK cells leaving the TME remains a rare event [173]. Importantly, NK cell activity can be inhibited when they perform trogocytosis. For example, after acquiring PD-1 by trogocytosis, NK cell anti-tumor activity is inhibited [174]. Moreover, in patients with breast cancer, the proportion of NK cells that have acquired HLA-G by trogocytosis is increased. These NK cells express also IL-10 and TGF-β that could impair their anti-tumor immunity efficiency [175]. NK cell enumeration and characterization provide information on tumor development and prognosis and also on treatment efficacy due to NK cell anti-tumor role. In a clinical trial on 54 patients with advanced NSCLC who received anti-PD-L1 immunotherapy, the group with higher NK cell activity showed a better median progression-free survival [176]. Recently, a retrospective study on patients with breast cancer who received neoadjuvant chemotherapy found that NK cell count was positively correlated with overall survival. Moreover, multivariate analyses showed that NK cell count was an independent predictor of neoadjuvant chemotherapy efficacy [177]. Similarly, the multivariate analysis of data from patients with NSCLC before and after second-line therapy with anti-PD-1/PD-L1 antibodies found that NK cells were the only lymphocyte type the number of which was a positive predictor of progression-free survival and overall survival [178]. Moreover, NK cell count appears to be predictive of the efficacy of a therapeutic vaccine in combination with first-line chemotherapy in patients with NSCLC [179]. In this study, outcome was worse in patients with higher numbers of CD16^+^CD56^+^CD69^+^ lymphocytes (*i.e.* activated NK cells). The authors stressed that in some contexts, NK cells hinder the adaptive response by secreting immunosuppressive cytokines, such as IL-10, and also by directly mediating their cytotoxicity towards macrophages or dendritic cells [179].

NK cells, as the very first line of immunological defense, are critical for eliminating tumor cells. In agreement, a prospective cohort study found that cancer incidence is lower in humans with high natural cytotoxic activity of peripheral blood lymphocytes [180]. Increased NK cell infiltration and high NK cell levels in the bloodstream have been frequently associated with better cancer outcome. Therefore, their quantification could become a biomarker for determining the success of treatments, such immunotherapies. Monitoring NK cells could also help to adapt therapeutic strategies and predict the patient response [181]. However, the interactions between NK cells and CTCs are still poorly understood. It is thought that NK cells can kill CTCs before their metastatic implantation; however, the exact mechanisms are unclear. For instance, it is not known whether NK cells can always remove CTCs, or whether CTCs can escape through complex strategies. This issue is critical for understanding how CTCs elude immune monitoring and for developing therapeutic strategies to increase NK cell activity against CTCs.

#### Links between CTCs and NK cells

Many CTCs can be released in the circulation, but only few will initiate secondary tumors at distant sites. These CTCs can survive in the new hostile environment represented by the bloodstream, and form a more aggressive subgroup that has developed or acquired the means to evade the immune system, particularly innate immune cells such as NK cells.

The first study on NK cell role in controlling CTC spread was published in 1980 [182]. The authors evaluated NK cell impact on the dissemination of circulating tumor emboli, which are aggregates of CTCs and stromal components, such as immune cells or fibroblasts. These clusters have a greater capacity to survive in the bloodstream and are more likely to form metastatic foci. In this study, cancer cells were inoculated in the bloodstream of mice and their dissemination to the lungs was analyzed in function of NK cell activity level. The number of lung metastases was higher in mice treated with cyclophosphamide, an immunosuppressive and immunomodulatory agent that decreases NK cell activity, compared with control mice. Adoptive transfer of spleen non-T cells in cyclophosphamide-treated mice reduced tumor dissemination to the level observed in controls. The authors concluded that the rapidly induced cytotoxicity (12—24 h) was due to NK cells and that they play a prominent role in controlling CTC dissemination [182]. More recently, Lo et al*.* showed that NK cells better control monoclonal metastasis because they preferably eliminate single CTCs rather than CTC clusters that are more resistant [183].

CTCs should be killed by NK cells, but they can evade this surveillance. For instance, IDO expression affects NK cell activity by modulating the expression of its activating receptors, such as NKp46 and NKG2D [102, 184]. Papadaki et al*.* demonstrated IDO expression on CTC surface in patients with NSCLC treated with anti-PD-1 immunotherapy. They suggested that CTCs displaying the IDO^+^ PD-L1^−^ phenotype may be of particular relevance for patient prognosis [185] because CTCs could use IDO expression to escape NK cell-mediated lysis. In a model of TGF-β-induced epithelial-mesenchymal transition, the NK cell inhibitory Killer Cell Lectin Like Receptor G1 (KLRG1) recognizes E-cadherin in tumor cells [161]. Moreover, the cytotoxic and regulatory T cell molecule (CRTAM), an NK cell-activating receptor, recognizes cell adhesion molecule 1 (CADM1). Thus, decreased E-cadherin and increased CADM1 expression levels enhance metastatic cell recognition by NK cells. This may partially explain how metastatic cells are recognized, although losing the inhibitory E-cadherin-KLRG1 interaction is not sufficient to restore NK cell cytotoxicity [161]. Hence, CTC control by NK cells probably depends on multiple receptors. Interestingly, NK cells also control dormant tumor cells that can give rise to cancers long after the primary tumor has been eliminated [186]. The expression of specific markers on CTC surface is one, but not the only way of evading NK cell surveillance. Indeed, CTCs can travel in the circulation with various cell types, including platelets. Many studies indicated that reduced platelet counts, a phenomenon known as thrombocytopenia, reduces tumor dissemination by improving NK cell anti-tumor surveillance [187, 188]. By binding to CTCs, platelets prevent the steric recognition by NK cells. Moreover, in a co-incubation experiment between colon cancer cells and platelets, platelets were activated and secreted TGF-β, thereby downregulating NKG2D expression [189]. Although this experiment was not performed with CTCs, it paves the way for better understanding the possible role of platelets in NK cell activity modulation in the context of tumor dissemination. Single-cell RNA sequencing data showed that patients with CTCs expressing platelet-related genes have worse prognosis [190]. Recently, in vitro, in vivo and ex vivo assays demonstrated that platelet adhesion to CTCs leads to increased CD155 expression. Upon binding to the inhibitory receptor TIGIT, CD155 inhibits NK cell cytotoxicity [191]. Liu et al*.* reported that in pancreatic ductal adenocarcinoma, CTCs can acquire the *RGS18* mRNA when interacting with platelets. This mRNA promotes the overexpression of the HLA-E complex, which through its interaction with the NKG2A/CD94 receptor inhibits NK cell cytotoxicity [192]. It is now acknowledged that platelets are involved in supporting tumor dissemination, and evidence is accumulating that they are associated with CTC evasion from NK cell immunosurveillance.

The role of NK cells in controlling tumor dissemination and eliminating CTCs is crucial in some cancer types [183]. Thanks to their ability to recognize and destroy tumor cells, they play a central role in preventing metastasis. Ichise et al*.* observed that after injection in mice, the number of melanoma cells disseminated to the lungs decreased rapidly in an NK cell-dependent manner [193]. In models of melanoma metastases to the lung, NK cells clear intravenously injected tumor cells, particularly when they pass through the liver [194]. These findings are clinically relevant; however, data on the immune evasion mechanisms used by CTCs to escape NK cell activity are lacking. More studies on these complex interactions are crucial to improve immunotherapy efficacy [163, 195–197].

### Clinical application of CTCs in NK cell-based therapy

NK cells play a vital role in controlling tumor dissemination, and CTCs develop mechanisms to evade them. Moreover, the detection and characterization of circulating NK cells and CTCs provide information on patient prognosis and treatment efficacy. Therefore, CTC analysis by liquid biopsy could be used to monitor the efficacy of NK cell-based therapies.

In a trial on 31 patients with NSCLC, CTCs were used to monitor the efficacy of NK cell-based therapy [198]. Peripheral blood samples were collected 1 day before treatment and then 7 and 30 days after injection. The mean number (± standard deviation) of CTCs was 27.12 ± 9.286 before injection and 14.02 ± 5.872 at day 30 after injection. This correlated with the presence of CTC-related RNAs in blood, such as CEA, CK18 and MAGE-3. The authors hypothesized that the decrease in CTC number correlated with a reduction in tumor mass and CTC release in the blood. They suggested CTC detection/enumeration as a candidate tool for determining the efficacy of allogeneic NK cell therapy [198].

It seems clear that CTC number is correlated with the presence of circulating lymphocytes. This was demonstrated in a study on 83 patients with late stage NSCLC [199] where the number of CTCs and of circulating lymphocytes (CD3^+^, CD4^+^, CD4^+^/CD8^+^ cells, and NK cells) were compared. CTC number was negatively correlated with NK cell number and this correlation was more pronounced in patients with stage IV than stage III NSCLC [199]. CTC detection and analysis may represent a promising tool for predicting the efficacy of NK cell-based cancer therapies. Monitoring CTC decrease after treatment could allow the indirect assessment of tumor mass reduction and cancer cell destruction. Thus, CTCs could become a dynamic biomarker for adjusting treatments in real time and optimizing therapeutic strategies to boost NK cell activity, and ultimately improve patient prognosis.

## Conclusion

This review highlights the crucial role of NK cells in immune surveillance and tumor control, showing their ability to eliminate cancer cells in the TME, in the lymphatic system and also in the bloodstream. Particularly, the key role of circulating NK cells in detecting and eliminating CTCs could limit metastatic dissemination. Their activity is often hindered by cancer-related mechanisms, and their counts and phenotypic characterization offer valuable prognostic information. In the future, CTCs could be included in liquid biopsy analyses as biomarkers to predict the efficacy of NK cell-based therapies, offering a promising avenue for adjusting therapeutic strategies. However, major gaps remain in our understanding of the interactions between CTCs and NK cells. The precise mechanisms by which CTCs manage to evade NK cell immune surveillance are still poorly known. This question should be better investigated because it could pave the way for new therapeutic approaches to better exploit NK cell potential for metastatic cancer treatment.

## Data Availability

No datasets were generated or analysed during the current study.

## Abbreviations

DCs: Dendritic cells
CTCs: Circulating tumor cells
HLA: Human leukocyte antigen
IDO: Indoleamine 2,3-dioxygenase
IFN: Interferon
KIR: Killer immunoglobulin-like receptors
MIC-A/B: MHC class I polypeptide-related sequence A and B
MHC: Major histocompatibility complex
NK cells: Natural killer cells
NSCLC: Non-small cell lung cancer
PD-1: Programmed cell-death protein 1
TGF-β: Transforming growth factor β
TINK: Tumor-infiltrating NK cells
TME: Tumor microenvironment
TNF: Tumor necrosis factor
T reg cells: Regulatory T cells
